# Effect of Resveratrol on Blood Rheological Properties in LPS-Challenged Rats

**DOI:** 10.3389/fphys.2018.01202

**Published:** 2018-08-29

**Authors:** Ying Wang, Hao Cui, Fei Niu, Shuo-Lin Liu, Yao Li, Li-Min Zhang, Hui-Bo Du, Zi-Gang Zhao, Chun-Yu Niu

**Affiliations:** ^1^Institute of Microcirculation, Hebei North University, Zhangjiakou, China; ^2^First Affiliated Hospital, Hebei North University, Zhangjiakou, China

**Keywords:** resveratrol, lipopolysaccharide, rheology, regional blood flow, intraperitoneal injection

## Abstract

**Objectives:** Abnormal rheological properties induce adverse effects during sepsis. This study sought to investigate the hypothesis that resveratrol (Res) improves blood rheological properties in rats following a lipopolysaccharide (LPS) challenge, and provide a novel approach for treatment of sepsis.

**Methods:** The rats were intraperitoneally or intramuscularly injected with vehicle, LPS (8 mg/kg), Res (30 mg/kg), or both to yield four groups: control, Res, LPS, and LPS + Res. After 6 h of LPS and/or Res injection, the mean arterial pressure (MAP), regional blood flow, erythrocyte and leukocyte parameters, and blood viscosity were observed.

**Results:** LPS administration had no significant effects on the erythrocyte parameters and plasma viscosity. LPS administration reduced the MAP, whole blood viscosity at low and medium shear rates, the blood flow in the spleen and kidney, and the leukocyte content in whole blood when compared to control group, and increased the myeloperoxidase (MPO) activity in lung. Treatment with Res alone had no effects on most of parameters observed except increasing the whole blood relative viscosity. However, Res treatment after LPS resulted in further decrease in whole blood viscosity at high and medium shear rates. Furthermore, Res treatment conversely decreased the red blood cell distribution width-CV, blood flow of stomach, whole blood relative viscosity and MPO activity in lung, and increased the leukocyte content, but did not restore LPS-induced decrease in MAP and the blood flow in the spleen and kidney.

**Conclusion:** The Res treatment partly reduce the whole blood viscosity and regional blood flow, and increase WBC content in peripheral blood following the LPS challenge, suggesting a favorable role in expanding the quasi-sympathetic effects of LPS in blood viscosity at early stages.

## Introduction

Sepsis induced by peritonitis is a common but critical pathological process (Ge et al., 2016). During sepsis, refractory hypotension, extensive microthrombus, abnormal hemodynamics, and microcirculatory disturbance lead to organ dysfunction and damage and/or death (Angus and van der Poll, 2013; Maloney, 2013). Numerous studies have shown that pre-treatment or post-administration of resveratrol (Res) could alleviate organ injury in animal models of endotoxic shock or sepsis induced by cecal ligation and puncture (Kolgazi et al., 2006), or administration of lipopolysaccharide (LPS) by either intravenous injection (Sebai et al., 2011), intraperitoneal injection (Zhao et al., 2014), or airway instilment (Knobloch et al., 2011). These beneficial effects of Res treatment are associated with anti-inflammatory response and relief from oxidative stress. Abnormal rheological properties play an important role during organ injuries induced by endotoxic shock (Lundy and Trzeciak, 2011; Teboul and Duranteau, 2012). Whether Res treatment improves abnormal rheological properties during sepsis induced by abdominal bacterial infections has not been reported. Considering these observations, we hypothesized that LPS administration induce the abnormal erythrocyte and leukocyte parameters, and further changes blood rheological properties. Therefore, the aim of the current study was to observe the effect of Res on blood rheological properties in rats following an LPS challenge via intraperitoneal injection.

## Materials and Methods

### Animals and Grouping

This study used 24 healthy and specific pathogen-free male Wistar rats that were purchased from the Chinese Academy of Medical Sciences Animal Breeding Center (Beijing, China). The rats, which weighing 170–210 g, were fasted for 12 h and were only allowed water before the start of the experiment procedures. The rats were randomly divided into the following four groups (*n* = 6): control, Res, LPS, and LPS + Res. All the procedures involving the animals were reviewed and approved by the Hebei North University Animal Care Committee and conformed to the guidelines of the National Institutes of Health. All efforts were made to minimize the suffering of the animals.

### LPS Challenge and Res Treatment

The LPS and Res administration were performed in conscious rats. First, the LPS and Res groups of rats were given intraperitoneal injection of LPS (5 mg/mL, 8 mg/kg; *Escherichia coli* O111:B4; Sigma, Milwaukee, WI, United States) and intramuscular injection of Res (300 mg/mL, 30 mg/kg; Sigma), respectively. In the LPS + Res group, an hour after the LPS administration, the Res treatment was performed. Vehicle injections into a group of rats were also conducted as a control [vehicles: saline for LPS and dimethylsulfoxide (DMSO, 100 μL/kg) for Res].

### Observation of Mean Arterial Pressure and Regional Blood Flow

At 4.5 h following the Res or DMSO administration, all the rats were anesthetized with pentobarbital sodium (1%, 50 mg/kg; Merck, Germany) and restrained in supine position. The right femoral operation was performed, and the femoral artery was separated and cannulated for the continuous monitoring of the mean artery pressure (MAP) levels of the animals for 10 min using the PowerLab biological signal collection and processing system (ML818, ADInstruments, Australia). Subsequently, the abdominal operation was performed to observe the regional blood flow of the liver, stomach, spleen, intestine, and kidney with a laser speckle flow monitoring system (PeriCam PSI, Perimed, Sweden). The blood flow images of the multiple organs were obtained using the image collection system of PSI, and the blood flow volume was obtained and shown as perfusion units (PU) using the data analysis system of PSI.

### Examination of Erythrocyte and Leukocyte Parameters and Blood Viscosity

After the observation of the regional blood flow, neck surgery was performed, and the common carotid artery was separated and cannulated to harvest the whole blood sample for the examination of erythrocyte and leukocyte parameters and blood viscosity.

Part of the blood sample of approximately 0.1 mL was fixed in an EP tube containing EDTA⋅K_2_ to examine the erythrocyte and leukocyte parameters, including the red blood cell content (RBC), hemoglobin (Hb), hematocrit (Hct), mean corpuscular volume (MCV), mean corpuscular hemoglobin (MCH), mean corpuscular-hemoglobin concentration (MCHC), red blood cell distribution width (RDW), white blood cell (WBC) content, and percentage and absolute value of neutrophil and lymphocyte, using a fully automatic laser blood corpuscle count analyzer (CA800, Sysmex, Japan) within 2 h. The 3.0 mL of heparinized arterial whole blood were loaded onto the sensing slot via straw under negative pressure conditions, and the whole blood viscosity at different shear rates of 10, 60, and 150 /s were analyzed using a hemorheological analyzer (LBY-N6Compact, Beijing, China). Then, the plasma sample was prepared through centrifugation at 850 × *g* for 10 min to determine the plasma viscosity. Finally, the whole blood relative viscosity and RBC aggregation index were calculated using the analysis system parameters of the hemorheological analyzer according to the whole blood viscosity, plasma viscosity, and Hct.

### Measurement of Intercellular Adhesion Molecule 1 (ICAM-1) Levels and Myeloperoxidase (MPO) Activities in Tissues

After harvesting blood sample, the lung, kidney, and myocardium were obtained from each rat, and homogenized in 1:9 (w/v) normal saline for 30 s for the preparation of homogenate. Then, the pulmonary, renal, and myocardial ICAM-1 levels and MPO activities were measured by ELISA method or hydrogen peroxide method, respectively, according to the manufacturer’s protocol.

### Statistical Analysis

All the obtained data were expressed as mean ± standard deviation (SD), and the statistical analysis was performed using SPSS software version 16.0 (SPSS Inc., Chicago, IL, United States). The differences among the experimental groups were analyzed via one-way analysis of variance, followed by Student–Newman–Keuls test. The values of *P* < 0.05 were considered as statistically significant.

## Results

### Effects of Res on MAP in Rats Following the LPS Challenge

**FIGURE 1:**
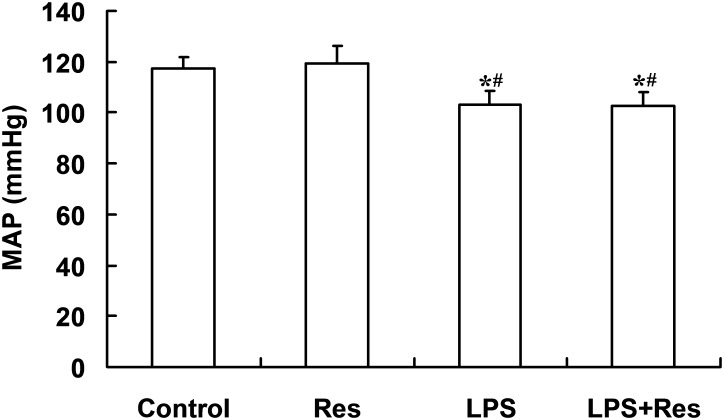
Effects of resveratrol (Res) on mean arterial pressure (MAP) in rats following lipopolysaccharide (LPS) challenge. Data are presented as the mean ± SD (*n* = 6). ^∗^*P* < 0.05 vs. Control group and ^#^*P* < 0.05 vs. Res group.

The intraperitoneal LPS stimulation significantly decreased the MAP levels compared to the control group (*P* < 0.05). By contrast, the Res treatment has no effect on the MAP and also did not restore the decreased MAP in the rats following the LPS injection (*P* > 0.05, **Figure 1**).

### Effects of Res on the Erythrocyte Parameters in Rats Following the LPS Challenge

The LPS injection did not result in statistical differences in the erythrocyte parameters (*P* > 0.05, **Table 1**). The LPS + Res group showed lower RDW-CV compared with the Res group, and higher MCH compared with the LPS group (*P* < 0.05), respectively (**Table 1**). There were no differences in the other erythrocyte parameters among all of groups (*P* > 0.05, **Table 1**).

**Table 1:** Effects of resveratrol (Res) on the erythrocyte parameters in rats following lipopolysaccharide (LPS) challenge (mean ± SD, *n* = 6).

Group	RBC (10^12^/L)	Hb (g/L)	Hct (%)	MCV (fl)	MCH (PG)	MCHC (g/L)	RDW-SD	RDW-CV	RBC aggregation index
Control	7.39 ± 1.04	152.83 ± 24.34	49.08 ± 5.90	66.63 ± 2.07	20.63 ± 0.55	310.50 ± 17.24	33.78 ± 1.92	16.12 ± 1.14	2.76 ± 0.76
Res	7.67 ± 0.66	157.00 ± 15.52	49.33 ± 2.16	64.33 ± 2.16	20.45 ± 0.43	318.33 ± 12.60	33.20 ± 2.23	16.70 ± 1.00	2.70 ± 0.28
LPS	7.36 ± 0.85	149.33 ± 15.40	47.58 ± 4.00	64.92 ± 2.75	20.32 ± 0.36	313.50 ± 9.18	31.95 ± 1.05	15.42 ± 2.09	2.54 ± 0.11
LPS + Res	7.00 ± 0.45	146.00 ± 7.64	46.00 ± 2.50	65.75 ± 1.78	20.88 ± 0.35^#^	317.50 ± 4.76	31.45 ± 1.52	14.35 ± 1.65^∗^	2.58 ± 0.43


*RBC, red blood cell; Hb, hemoglobin; Hct, hematocrit; MCV, mean corpuscular volume; MCH, mean corpuscular hemoglobin; MCHC, mean corpuscular hemoglobin concentration; RDW, red blood cell distribution width. ^∗^*P* < 0.05 vs. Res group and ^#^*P* < 0.05 vs. LPS group.*

### Effects of Res on the Blood Viscosity in Rats Following the LPS Challenge

**FIGURE 2:**
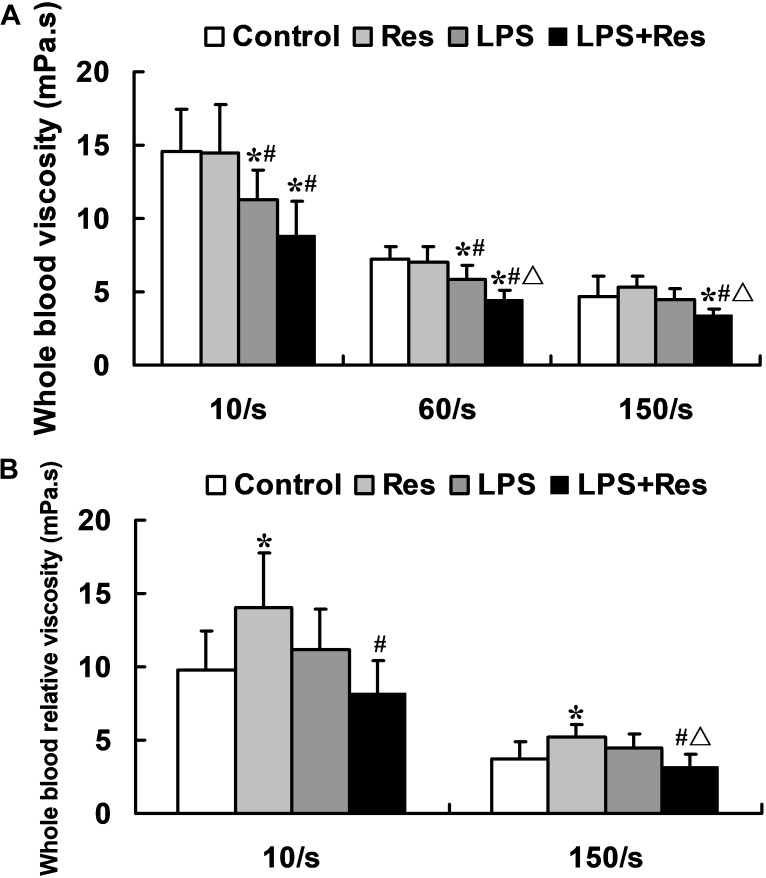
Effects of resveratrol (Res) on whole blood viscosity indices in rats following lipopolysaccharide (LPS) challenge. Data are presented as the mean ± SD (*n* = 6). **(A)** Whole blood viscosity; **(B)** Whole blood relative viscosity. ^∗^*P* < 0.05 vs. Control group; ^#^*P* < 0.05 vs. Res group; and ^Δ^*P* < 0.05 vs. LPS group.

The LPS injection significantly reduced the whole blood viscosity at low and medium shear rates (*P* < 0.05), but no difference at a high shear rate (*P* > 0.05, **Figure 2A**). In contrast, Res alone had no effect on the whole blood viscosity, but further enhanced the LPS-induced decrease in the whole blood viscosity (*P* < 0.05, **Figure 2A**). The LPS had no effect on the whole blood relative viscosity while Res alone increased this parameter (*P* < 0.05, **Figure 2B**). Surprisingly, Res treatment resulted in a reduced effect on the whole blood relative viscosity under the LPS challenge (*P* < 0.05, **Figure 2B**). Moreover, no statistically significant difference was found in the plasma viscosity at 150 /s among the Control, Res, LPS, and LPS + Res groups (1.31 ± 0.38, 1.04 ± 0.08, 1.02 ± 0.10, and 1.13 ± 0.47 mPa s, respectively) (*P* > 0.05).

### Effects of Res on the Regional Blood Flow in Rats Following the LPS Challenge

**FIGURE 3:**
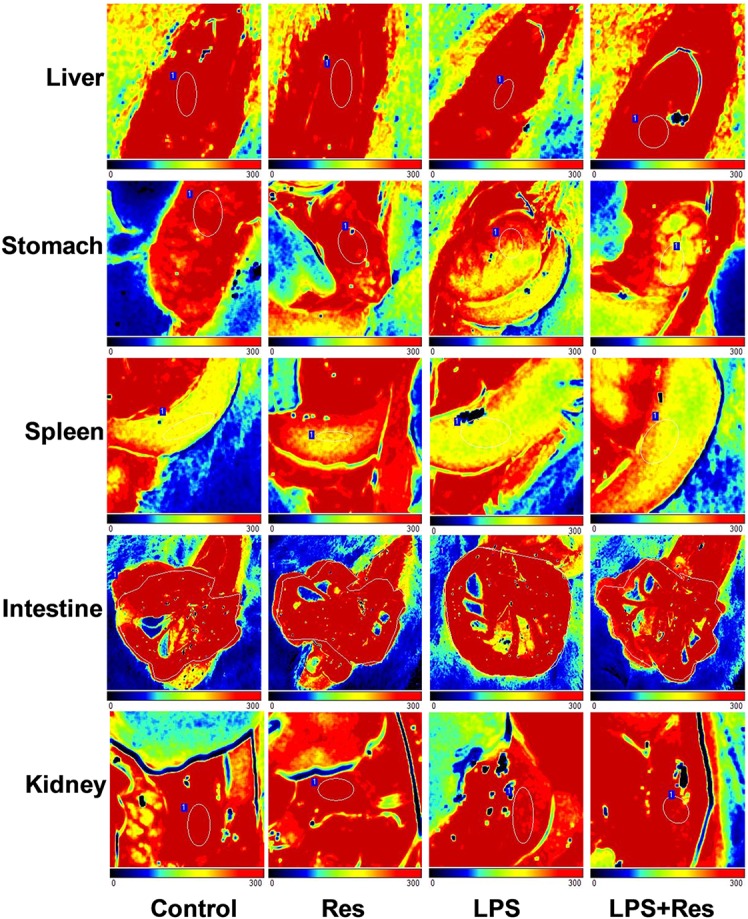
Effects of resveratrol (Res) on blood flow volume of multiple organs in rats following lipopolysaccharide (LPS) challenge.

The intraperitoneal LPS injection decreased the blood flow in the spleen and kidney compared with the control group (*P* < 0.05, **Table 2** and **Figure 3**). However, it did not obviously affect the blood flow in the liver, stomach, and intestine (*P* > 0.05). Res did not restore the attenuated blood flow in the spleen and kidney, even reduced the blood flow of stomach under LPS stimulation.

**Table 2:** Effects of resveratrol (Res) on blood flow volume (perfusion units, PU) of organs in rats following lipopolysaccharide (LPS) challenge (mean ± SD, *n* = 6).

Group	Liver	Stomach	Spleen	Intestine	Kidney
Control	396.6 ± 52.3	308.5 ± 82.8	195.2 ± 47.3	216.0 ± 67.9	411.6 ± 57.5
Res	410.5 ± 27.8	340.3 ± 45.3	207.8 ± 27.8	225.5 ± 37.2	424.7 ± 57.8
LPS	397.3 ± 76.1	307.8 ± 92.3	154.5 ± 15.7^#^	204.7 ± 40.8	314.4 ± 66.3^∗#^
LPS + Res	442.9 ± 47.6	196.4 ± 57.4^∗#Δ^	174.8 ± 61.2	221.2 ± 25.6	323.4 ± 58.4^∗#^


*^∗^*P* < 0.05 vs. Control group; ^#^*P* < 0.05 vs. Res group; and ^Δ^*P* < 0.05 vs. LPS group.*

### Effects of Res on the Leukocyte Parameters in Rats Following the LPS Challenge

**FIGURE 4:**
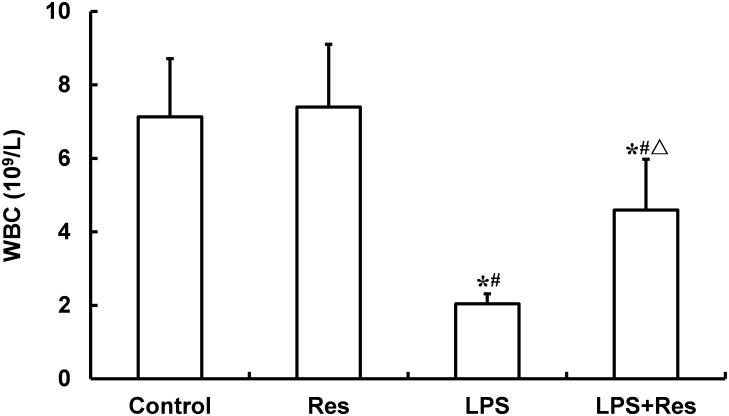
Effects of resveratrol (Res) on white blood cell (WBC) content (10^9^/L) in peripheral blood in rats following lipopolysaccharide (LPS) challenge. Data are presented as the mean ± SD (*n* = 6). ^∗^*P* < 0.05 vs. Control group; ^#^*P* < 0.05 vs. Res group; and ^Δ^*P* < 0.05 vs. LPS group.

The LPS injection reduced the WRC content and absolute value of neutrophil and lymphocyte in peripheral blood comparted to the control and Res group (*P* < 0.05, **Figure 4** and **Table 3**). The Res treatment enhanced the WRC content and lymphocyte absolute value, but decreased the lymphocyte percentage, in peripheral blood in rats subjected to LPS challenge (*P* < 0.05, **Figure 4** and **Table 3**). In addition, there were no statistical differences in the other indices among these groups (*P* > 0.05).

**Table 3:** Effects of resveratrol (Res) on the indices of white blood cell in rats following lipopolysaccharide (LPS) challenge (mean ± SD, *n* = 6).

Group	Percentage of neutrophil (%)	Percentage of lymphocyte (%)	Neutrophil (10^9^/L)	Lymphocyte (10^9^/L)
Control	17.13 ± 10.23	80.58 ± 11.56	1.33 ± 0.88	5.87 ± 1.06
Res	12.75 ± 6.41	81.38 ± 3.10	0.92 ± 0.44	6.26 ± 1.14
LPS	9.96 ± 6.98	76.92 ± 8.48	0.20 ± 0.17^∗#^	1.51 ± 0.23^∗#^
LPS + Res	13.34 ± 5.92	64.47 ± 7.13^∗#Δ^	0.46 ± 0.16^∗^	2.40 ± 0.50^∗#Δ^


*^∗^*P* < 0.05 vs. Control group; ^#^*P* < 0.05 vs. Res group; and ^Δ^*P* < 0.05 vs. LPS group.*

### Effects of Res on the ICAM-1 Levels and MPO Activities in Rats Following the LPS Challenge

**FIGURE 5:**
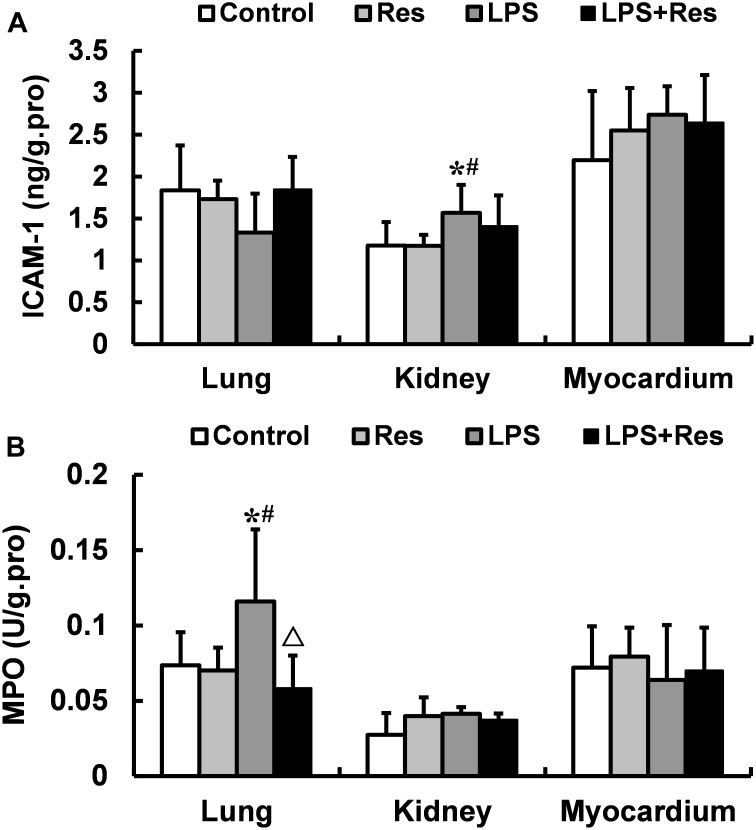
Effects of resveratrol (Res) on the intercellular adhesion molecule 1 (ICAM-1) levels and myeloperoxidase (MPO) activities in lung, kidney, and myocardium in rats following lipopolysaccharide (LPS) challenge. Data are presented as the mean ± SD (*n* = 6). **(A)** ICAM-1 levels; **(B)** MPO activities. ^∗^*P* < 0.05 vs. Control group; ^#^*P* < 0.05 vs. Res group; and ^Δ^*P* < 0.05 vs. LPS group.

The LPS challenge enhanced the ICAM-1 level in rat’s kidney and the MPO activity in rat’s lung comparted with the control and Res group, and the pulmonary MPO activity was abolished by the Res treatment (*P* < 0.05, **Figure 5**). However, there were no differences in the MPO activities in kidney and myocardium among these groups (*P* > 0.05).

## Discussion

To reveal the mechanism underlying peritonitis-induced sepsis and multiple organ injuries and to find the effective countermeasures for the sepsis, an LPS dose of 5–120 mg/kg has been constantly used to establish the model of the LPS challenge through intraperitoneal injection. Li et al. (2011) reported that the intraperitoneal injection of a large dose of LPS (35 mg/kg) resulted in the death of all C57B1/6J mice within 23 h (100% mortality). Whereas the administration of a small dose of LPS (10 mg/kg) significantly reduced the mortality rate (10% mortality). The LPS (1 mg/kg) caused hypothermia, which occurred 20 min after the intraperitoneal injection; while the high dose of LPS (60 mg/kg) caused profound hypothermia (Akyol et al., 2008). These results indicated the positive correlation between LPS dose and the severity of sepsis. In order to observe the evolution process of sepsis patients and with consideration of the differences in surface area between the rats and mice, 8 mg/kg of LPS was injected intraperitoneally into the rats for the investigation of the putative effect of Res on rheological properties in the present experiments.

It is well established that the MAP level reflects the hemoperfusion status of body, which is related to circulatory function. Recently, Lorigados et al. (2015) reported that the intraperitoneal injection of 10 mg/kg LPS induced a hypotension with MAP ≤ 65 mmHg in rats. In our previous investigation, the intraperitoneal injection of 35 mg/kg LPS into the mice resulted in hypotension that was approximately 70% below the normal level. In contrast to these previous studies, we found that the LPS injection decreased the MAP levels by 10% in this study. This result indicates that the low dose of LPS challenge in the healthy rats resulted in a slight decrease in the blood pressure that was on behalf of a mild abdominal infection. The results from the work of Staehr et al. (2013) showed that LPS bolus led to significant decreases in MAP (by approximately 30 mmHg) and heart rate (50% of baseline); moreover, the continuous infusion of cyclosporine A (CsA, 20 or 40 mg/kg/24 h), which is similar to Res, did not demonstrate an obvious effect on MAP and heart rate at 0–24 h after LPS administration. In the present work, the Res treatment had no obvious effect on the MAP levels of the rats subjected to LPS challenge, which was similar with the role of CsA in the previous study (Staehr et al., 2013).

Whole blood viscosity is associated with RBC counts, Hct, RBC aggregation and deformability, and so on (Csorba et al., 2013; Viallat and Abkarian, 2014), which is an important factor to reflect hemodynamics. The previous study have shown that the intraperitoneal LPS injection reduced the RBC deformability, decreased the nitric oxide production in RBCs, and inhibited the activation of the Na,K-ATPase in erythrocyte membranes (Radosinska et al., 2016). However, whether LPS administration plays a role on erythrocyte counts, Hct, and MCV, etc., it is unclear. In the present study, we found that the intraperitoneal LPS injection did not exhibit obvious effects on these indices, including RBC, Hb, Hct, MCV, MCH, MCHC, RDW-SD, RDW-CV, and RBC aggregation index. This result might be related to the low dose of LPS. The Res treatment induced a declining trend in Hct, suggesting that Res plays a role in hemodilution, which is beneficial to improve microcirculation and blood flow. Res also increased the MCH count at a favorable level that would enhance the oxygen carrying capacity of a single erythrocyte. In conclusion, the Res treatment played a certain role in the LPS administration. However, the long-term effect of Res on erythrocyte parameters after an LPS challenge requires further investigation.

Blood viscosity is an important factor in microcirculation and hypoperfusion (Guerci et al., 2014; Song et al., 2014). Our data showed that the intraperitoneal LPS injection reduced the whole blood viscosity at low and medium shear rates; this result is in contrast to the effect of the LPS incubation to increase red cell membrane viscosity *in vitro* (Todd et al., 1993). In general, the increased blood viscosity by sepsis is unfavorable for the prognosis of severe patients. So, we think that the decreased whole blood viscosity by LPS challenge is a benign adaptive change, which is favorable for the body against LPS challenge. Therefore, such finding in the current study could be attributed to the sympathomimetic effect of LPS after its transfer to the blood circulation via vascular endothelial barrier, which induced vasoconstriction, increased the volume of interstitial fluid returning to the blood circulation, and resulted in hemodilution. However, the long-term effect of LPS attack or higher dose of LPS on whole blood viscosity requires further investigation. We further found that the Res treatment significantly decreased the whole blood viscosity at high, medium, and low shear rates; as well as the whole blood relative viscosity at a high shear rate in rats following the LPS challenge, which might enhance the LPS-induced sympathomimetic action. These results indicate that Res played an important role in activating blood circulation to dissipate stasis. As a result, the blood perfusion of the vital organs improved.

No statistically significant difference was found in the plasma viscosity at 150 /s among the Control, Res, LPS, and LPS + Res groups. In general, plasma viscosity relates to the protein level in plasma. LPS injection might decrease the level of acute phase protein. By contrast, the LPS injection in this study also induced hemodilution partly. The plasma protein level showed no obvious increase, which could therefore explain the lack of difference in the plasma viscosity.

The level of regional blood flow was directly used to evaluate the blood perfusion in the multiple organs. The current results showed that the intraperitoneal LPS injection decreased the blood flow in the spleen and kidney and that the Res treatment further decreased the blood flow in the stomach. Generally, abdominal organs, including spleen, kidney, intestine, stomach, and liver, have widely sympathetic nerves. Therefore, the quasi-sympathetic effects of LPS at an early stage induced the vasoconstriction of these organs and blood flow redistribution. These results are consistent with those for blood viscosity, which has a compensatory effect to improve microcirculation. The previous study showed that the LPS treatment decreased arterial liver perfusion after 5 h and induced the reduction of hepatic blood flow (Tamandl et al., 2008). However, the present research did not found that the decreasing effect of LPS injection on the hepatic blood flow. Therefore, the long-term changes in the regional blood flow subjected to the LPS challenge require further observation to understand the effects of Res.

After LPS administration, the leukocyte is related to not only inflammation, but also blood viscosity, especially LPS-induced leukocyte adhesion. So, we observed the changes of leukocyte parameters, and found that the intraperitoneal injection of LPS resulted in a decrease in leucocyte content, which is accordance with the previous study (Sebai et al., 2009). Furthermore, the Res treatment enhanced the WRC content in peripheral blood obtained to LPS treated rats. These data suggested that the beneficial effect of Res on LPS attacked rats might be related to inhibiting the reduction of WBC. However, what is the mechanism by which LPS deceasing WBCs? And where are the reduced leucocyte in peripheral blood after LPS administration, it remains further observation.

In previous studies, the intravenous injection of LPS induced a large number of WBCs adhesion to vascular endothelial cells in mesentery microvessel, and increased ICAM-1 level in plasma in rats (Zhang et al., 2011), Res treatment reduced the expressions of ICAM-1 and vascular cell adhesion molecule 1 (VCAM-1) in human microvascular endothelial cells subjected to LPS (Park et al., 2009). Therefore, we hypothesize that LPS administration increase the hyper expressions of cell adhesion molecules, and further induce the adhesion of leukocytes to endothelial cells and infiltration of leukocytes to tissues through the vascular endothelial barrier. To verify this hypothesis, we investigated that the effects of Res on the ICAM-1 level and MPO activity in tissues. The results showed that intraperitoneal injection of LPS increased the content of ICAM-1 in kidney and MPO activity in lung, and the Res intervention decreased the MPO activity in lung. The results demonstrated that the reduction of leucocyte was infiltrated to the lung tissue, which is a reason that LPS decreased leucocyte content. But, the mechanism of leucocyte infiltration is not related to ICAM-1 after LPS challenge, which is need to further investigation.

In summary, current results indicate that the quasi-sympathetic effects of the LPS within 6 h led to blood flow redistribution and hemodilution and further decreased the regional blood flow in the spleen and kidney, as well as the whole blood viscosity. However, the Res treatment partly reduced the whole blood viscosity and regional blood flow following the LPS challenge. This result favored the expansion of the quasi-sympathetic effects of the LPS at early stages. In addition, Res treatment plays an increase effect on leucocyte content in peripheral blood, which is related to the decrease in leucocyte infiltration. However, Res treatments must be investigated further to alleviate LPS-induced injuries. Given the high mortality rate and abnormal hemodynamics in patients with sepsis (Ge et al., 2016), the current results suggest that Res treatment may potentially play a protective role among sepsis patients.

## Author Contributions

Z-GZ and C-YN conceived and designed the work. YW, HC, FN, S-LL, YL, L-MZ, and H-BD acquired and analyzed the data for the work. YW wrote the manuscript. Z-GZ and C-YN critically revised the manuscript.

## Conflict of Interest Statement

The authors declare that the research was conducted in the absence of any commercial or financial relationships that could be construed as a potential conflict of interest.
